# Dichorionic twin pregnancy with sirenomelia and chromosomal anomaly in 1 fetus: A case report

**DOI:** 10.1097/MD.0000000000024229

**Published:** 2021-01-08

**Authors:** Yuan Ting, Li Xue-Lan, Wang Chun-Bao, Zhang Ting, Li Fen, Han Zhen

**Affiliations:** a Department of Obstetrics & Gynecology; b Department of Pathology, First Affiliated Hospital of Xi’an Jiaotong University, Shaanxi, China.

**Keywords:** chromosomal abnormality, sirenomelia, twin

## Abstract

**Rationale:**

Sirenomelia is a rare congenital malformation that threatens fetal survivals. The cases in which twin with sirenomelia and chromosomal abnormality have been seldomly reported. We reported a dichorionic twin case in which one twin had sirenomelia, the other twin had a normal phenotype, and they had different chromosomal abnormalities.

**Patient concerns:**

The abnormal twin was found at 22 weeks by ultrasound. The sirenomelia fetus was complicated with a thoracic stenosis, enlarged rectum without anal opening, the absence of bilateral kidneys, a single umbilical artery, a single lower limb, the abnormal curvature of spine, double outlet of right ventricle, which were the indicatives of the chromosome detection.

**Diagnosis:**

The copy number variation of the sirenomelia fetus was detected as a deletion of 4.8Mb in 11p11.12-11q11. The co-twin was found with del(Y)(q11.223q11.23), which was as the same as his father's. The mother had normal chromosome. The parents had normal phenotypes. It was firstly reported a microdeletion with sirenomelia fetus.

**Interventions:**

There was no specific treatments for the twins.

**Outcomes:**

Intrauterine death of the sirenomelia fetus was found at 27 weeks and postnatal death after inevitable abortion happened to the co-twin.

**Lessons:**

Prenatal ultrasound was responsible for recognizing sirenomelia, and the detailed ultrasound scanning and chromosome detection should be done for the co-twin. The etiology of sirenomelia remains unclear, and genetic detection is also necessary for its pathogenesis research.

## 1 Introduction

Sirenomelia is a rare congenital malformation, and its prevalence is estimated to vary between 1/600,000–1,000,000.^[1]^ The incidence of sirenomelia in twins is extremely low; however, the risk of sirenomelia in monozygotic twins is nearly 100 to 150 times higher than that of dizygotic twins and singletions.^[2]^ Sirenomelia is characterized by the fusion of the lower extremities, and renal agenesis, absent urinary tract and genitalia, imperforate anus, oligohydramnios, and a single umbilical artery.^[3]^ Sirenomelia is definitely a serious threat to fetal survival due to the associated severe malformations. More than 50% of sirenomelia cases result in stillbirths, and the live neonates with sirenomelia often die within 1 to 2 days after birth.^[4]^

Here, we present a case of dichorionic twin in which 1 twin had sirenomelia while the other twin had a normal phenotype, and both fetuses had different chromosomal abnormalities. To the best of our knowledge, chromosomal abnormality of the fetus with sirenomelia has not been reported in the literature.

## 2 Case presentation

A 29-year-old woman patient, gravida 2, para 1, who was a farmer, had a pre-pregnancy body mass index of 28.4 kg/m^2^ (pre-pregnancy weight of 70 kg and height of 157 cm). The patient's first child was female and was delivered vaginally at 37 weeks, and the birth weight was 2150 g. The child was prone to suffer from tonsil hypertrophy and upper respiratory tract infections.

The current twin gestation was spontaneously fertilized without fetal reduction management in early stage. Dichorionicity was finally demonstrated at the time of delivery. The patient had no history of chronic diseases (such as thyroid disease, hepatic disease, renal disease, endocrine disease, polycystic ovarian syndrome or anemia). Until the time of delivery, no obstetrical complications occurred (such as hypertensive disorder, diabetes mellitus, intrahepatic cholestasis or placenta previa).

**Figure 1:**
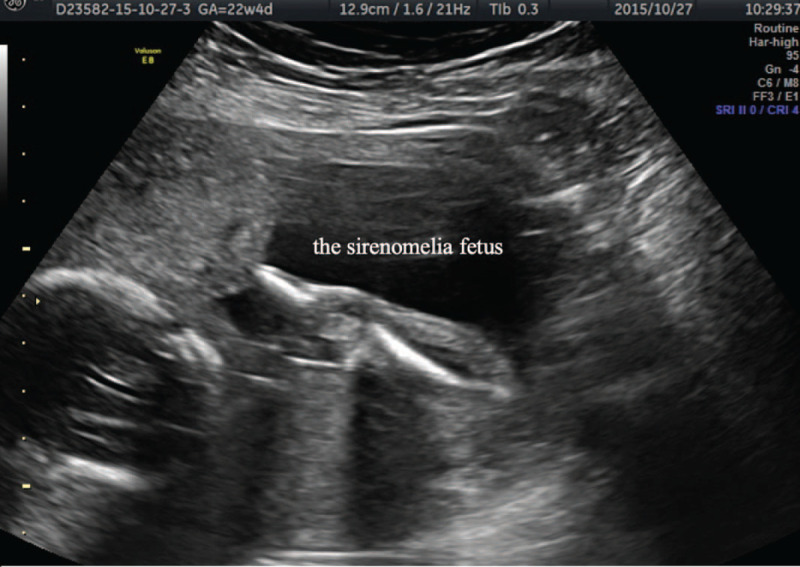
The fetus with sirenomelia with a single lower limb.

The original suspicion of sirenomelia was noted by 1 tertiary hospital (hospital 1) when the patient went for ultrasound examination at 22 weeks. Four days later, she came to our hospital for further diagnosis. The ultrasound showed that the fetus with sirenomelia (Fig. 1) was smaller than the other twin by 12 days and had oligohydramnios (no obvious amniotic pocket) and absence of end diastolic velocity in the umbilical artery. The fetus also had a series of abnormalities, including a narrow thoracic cavity, absence of bilateral kidneys and bladder, flexion deformation of the spine, double outlet of right ventricle (DORV), and a single umbilical artery. The co-twin showed no obvious structural abnormalities, and its deepest amniotic volume pocket was 6.9 cm.

**Figure 2:**
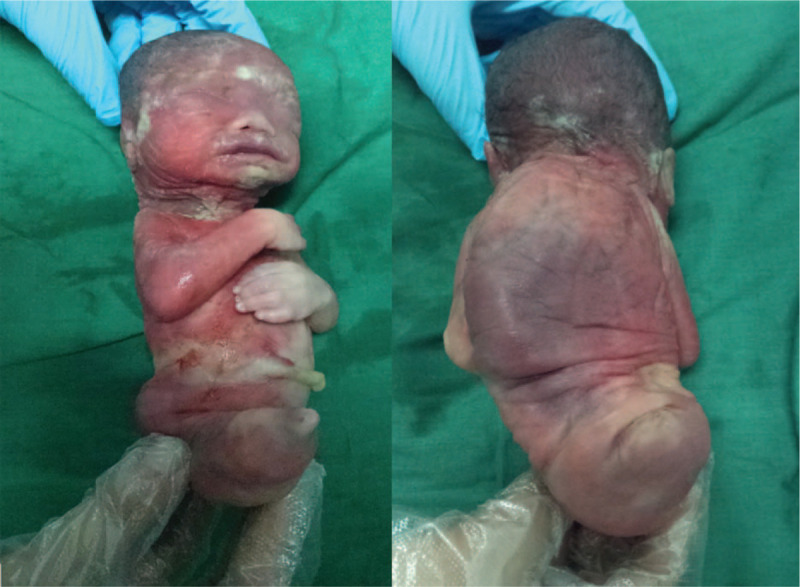
The fetus with sirenomelia after inevitable abortion.

Then, the patient went to another tertiary hospital (hospital 2) for amniocentesis for the co-twin. At the same time, the patient and her husband (father of the twins) also underwent peripheral blood testing for chromosome detection. The chromosomal detection was conducted by array-based Comparative Genomic Hybridization. The results of the father and the co-twin were the same and showed the chromosomal abnormality of del(Y)(q11.223q11.23). The patient and her husband decided to continue the pregnancy with the hope that the co-twin with a normal phenotype would survive. At 27^+4^ weeks, the patient was admitted in our hospital's emergency department due to threatened abortion. The fetus with sirenomelia was already dead (almost at 26 gestational weeks according to fetal growth measurements) (Fig. 2), as revealed by the ultrasound at admission. Then, inevitable abortion finally happened. The birth weight of the co-twin was 980 g and the Apgar score was 3, 3, and 6 for 1 minute, 5 minutes, and 10 minutes, respectively. No obvious abnormalities were found in its appearance. The co-twin was taken home by parents and soon died.

After acquiring consent from the patient, an autopsy was performed for the sirenomelia twin. The results were as follows: thoracic stenosis; an enlarged rectum without anal opening; a pair of kidney-like organs was located in the posterior peritoneum; a pair of testis-like tissue was located in the peritoneal cavity; a small bladder; a single lower limb extending from the middle to the right side of the body; the entire spine bending to the left side of the body; a flexion deformation of the right upper limb; the right fingers were overlapped strangely; cardiac malformation of DORV. The pathological examination finally demonstrated a single umbilical artery, the absence of bilateral kidneys, the presence of bilateral adrenal glands, and the presence of testis and epididymis. The sex of the fetus was male.

**Figure 3:**
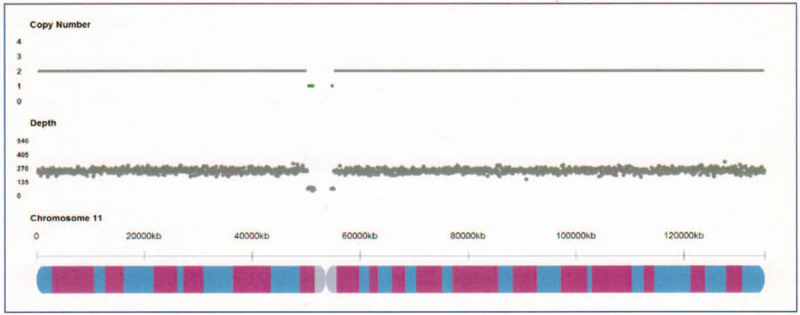
A deletion of 4.8Mb in 11p11.12-11q11 was found by copy number variation detection in the fetus with sirenomelia.

At the same time, a piece of skin tissue was obtained from the sirenomelia twin for chromosomal karyotyping and copy number variations detection and finally, del(11) (p11.12q11) (GRCh37: Chr11: 50260001-55117000) was detected, which was a deletion of 4.8Mb in 11p11.12-11q11 (Fig. 3).

## 3 Discussion

Sirenomelia is a rare congenital syndrome, that is lethal to fetal survival. The risk factors of sirenomelia include maternal diabetes mellitus, exposure to teratogenic drugs or agents^[5]^ (such as, retinoic acid, cyclophosphamide, or cadmium), and a maternal age less than 20 years or more than 40 years.^[3]^ In the current case, the patient had no diabetes and was pregnant at an appropriate age; however, it was difficult to determine whether the woman was exposed to certain chemical agents in her daily agricultural activities. To date, there are 2 hypotheses commonly regarding the pathogenesis. The vascular steal hypothesis^[6]^ proposes that sirenomelia arises from a deformed blood vessel that originates from the high abdominal aorta, which performs the function of the umbilical artery and transports a large amount of blood from the umbilical cord to the placenta; and the blood perfusion to the abdominal aorta is insufficient, eventually leading to severe malformations of the spine, lower limbs, and genitourinary system. The other hypothesis is the defective blastogenesis hypothesis,^[7]^ which proposes that damage to the caudal mesoderm of the embryo between 13 and 22 days in early life may result in the merging, malrotation, and dysgenesis of the lower extremities.

There are a series of characteristic sirenomelia phenotypes. A previous study has reported the frequencies for the following main malformations of sirenomelia (compiled by the results of 2 articles, the sample size was 87 cases)^[8]^: vertebrae/sacrum/pelvis/lower limb (100%), anorectal (97%), renal (93%), genital (85%), lower urinary tract (57%), single umbilical artery (79%), cardiac (26%), radial limb (21%), esophageal atresia or/and tracheoesophageal fistula (5%), respiratory tract (24%), and central nervous system (8%). Oligohydramnios is a common feature in the cases with sirenomelia due to the abnormal development of the renal system. However, Pinette MG et al^[9]^ reported a case where the fetus was initially found with oligohydramnios at 18 weeks, but the amniotic fluid index was 11 cm at 25 weeks.

In 21 previous studies,^[10–30]^ twins with sirenomelia were delivered between 27 and 38 weeks, except for 1 case that was terminated in the second trimester. The co-twins with normal anatomy almost had good outcomes (a co-twin with an imperforate anus was healthy after operation^[18]^). In the present case, intrauterine death of the twin with sirenomelia initially occurred, followed by spontaneous abortion. The co-twin eventually died despite normal anatomy. Based on the previous cases, it was speculated that the outcome of the co-twin without structural abnormalities might be good. With intensive prenatal monitoring, expectant management could be carried out for cases where the co-twin has normal anatomy and normal chromosome. If the sirenomelia happened in monochorionic case, prenatal monitoring of the co-twin should be enhanced when intrauterine death occurs to the sirenomelia twin. This is because the live fetus can be affected due to only 1 placenta shared by the twins and the acute transfusion by placental inter-twin anastomoses.^[31]^ Preterm birth is the most common adverse outcome affecting 58.5% of monochorionic twins. In total, 20% of the surviving co-twins were demonstrated to have abnormal brain alterations and the risk of perinatal death also increased in monochorionic twins.^[32]^ If the co-twin was found with severe structure abnormalities or detected with chromosomal abnormalities, we usually suggest not to continue the pregnancy. So a complete evaluation for the co-twin is important, which determines whether it is prudent to continue the pregnancy.

Among the previously reported cases, only 2 cases reported sirenomelia fetus with chromosomal abnormalities, including mosaicism of 69,XXX/46,XX and a de novo balanced translocation of 46,XX,t(X;16)(p11.23;p12.3).^[5,33]^ The other cases reported normal chromosome or without chromosome detection. To the best of our knowledge, the current case is the first to report a microdeletion of 4.8 Mb in 11p11.12-11q11 with sirenomelia fetus. It is not yet clear if sirenomelia in twins has a genetic basis.

The genes OR4A5, TRIM48, and ORA416 are located in the involved chromosome segment. The search for the segment and genes was performed in the databases of DECIPHER, DGV, ISCA, and OMIM databases (DECIPHER is a web-based resource and database of genomic variation data from analysis of patient DNA. DGV provided a publicly accessible, comprehensive curated catalog of structural variation found in the genomes of control individuals from worldwide populations. ISCA database contains whole genome array data from a subset of the ISCA Consortium clinical diagnostic laboratories. OMIM is a comprehensive, authoritative compendium of human genes and genetic phenotypes that is freely available and updated daily), and no related literature was found. Further studies to elucidate its underlying genetic mechanism are needed.

## 4 Conclusions

In summary, the prenatal ultrasound can help in recognizing sirenomelia, which is crucial due to the serious threat of sirenomelia to fetal survival, and a detailed ultrasound scanning and chromosome detection for the co-twin should be done, which may provide evidences for the possibility of survival of the normal twin. The etiology of sirenomelia remains unclear, and genetic detection is also necessary for to understand its pathogenesis.

## Acknowledgments

The authors would like to thank all of the members in the team involved in the study.

## Author contributions

**Data curation:** Chun Bao Wang.

**Funding acquisition:** Fen Li, Zhen Han.

**Investigation:** Xue Lan Li, Chun Bao Wang.

**Project administration:** Ting Yuan.

**Resources:** Fen Li.

**Supervision:** Ting Yuan, Xue Lan Li, Zhen Han.

**Writing – original draft:** Ting Yuan, Ting Zhang.

**Writing – review & editing:** Ting Yuan, Ting Zhang.
